# New Insights into Bacterial Pathogenesis

**DOI:** 10.3390/pathogens12010038

**Published:** 2022-12-26

**Authors:** Carmelo Biondo

**Affiliations:** Department of Human Pathology, University of Messina, Via C. Valeria n.1, Policlinico Universitario “G. Martino”, Gazzi, 98125 Messina, Italy; cbiondo@unime.it; Tel.: +39-0902213312

Pathogenicity, or the ability of a microorganism to cause disease, depends on several factors, among which the immune status of the host and the microbial species involved in the exposure play a key role. Microbial pathogenicity is a complex, multifactorial process, governed by genetic and molecular features associated with virulence and/or resistance determinants involved in critical steps of pathogenesis [1]. An estimated 60 percent of known human infectious diseases are caused by zoonotic transmission from domestic and wild animals, and an alarming 75 percent of all emerging infectious diseases are zoonotic in origin [2]. In addition, a considerable number of pathogenic diseases and transmission routes may be exacerbated by climate change, posing a serious threat to human health [3]. In the past two decades, several new emerging viruses have caused local outbreaks, epidemics, and pandemics characterized by high mortality rates and a high incidence of post-infection disorders [4]. The severe acute respiratory syndrome coronavirus 2 (SARS-CoV-2) pandemic has revealed the urgent need for the early identification of potential threats and the timely development of treatment and prevention measures [5]. It is known that, on the one hand, viral infection can predispose to bacterial disease just as, on the other hand, bacterial infection can facilitate systemic viral spread, and combined infections can exacerbate tissue damage [6]. The pathogenetic mechanisms of most bacterial diseases are still poorly understood. A major obstacle to the development of new drugs and vaccines is the limited knowledge of host–microbe interactions and the detailed processes by which infection is established. The mechanisms used by bacteria to cause disease in the host are varied and can be specific to a particular species or conserved across species [7]. This explains the diversity of infection modes, target cells, and molecular mechanisms underpinning bacterial pathogenesis. Many bacterial infections share common characteristics related to the acquisition of virulence and antibiotic resistance genes that can be spread by horizontal transfer through mobile genetic elements (e.g., plasmids and transposons) to other bacteria [8].

This Special Issue of *Pathogens*, titled "New Insights into Bacterial Pathogenesis," aims to collect recent findings regarding the relationship between virulence and resistance that allows pathogenic bacteria to colonize and invade human tissues. In this Special Issue, readers will find contributions (six experimental publications and three review articles) that provide information on various aspects concerning bacterial pathogenicity, such as mechanisms of pathogenicity, antibiotic resistance, strategies to maintain elements essential for bacterial growth and survival, and the application of molecular techniques for the identification of various pathogens.

The original manuscript by Swietnicki and colleagues is the first report in the literature to analyze in detail the O-type antigen biosynthetic pathway of a human oral pathogen involved in dental tissue destruction, *Porphyromonas gingivalis* [9]. They found the genes and pathways for O-type antigen biosynthesis using public information and computational analysis of whole genomes with the help of Pathway Tools software. The predicted pathway was identified through a combination of bioinformatic analysis and experimental data. The approach followed by these authors could also be used to identify biosynthetic pathways of other bacterial structures that could be targets for vaccine development.

Another original study conducted by Bonifácio et al. analyzed the natural transformation capacity of *Aliarcobacter butzleri*, an emerging enteropathogen that shows high genetic diversity [10]. The authors showed, for the first time, that horizontal gene transfer mediated by natural transformation is one of the key processes contributing to the genetic diversity of this microorganism. In addition, the authors developed a new protocol to improve molecular research on this microorganism, thus providing a useful tool for for its genetic manipulation.

Elhakim et al. are the authors of a pilot study that investigated the role of zinc-metal ions in the pathogenesis of *S. aureus* [11]. This work describes, for the first time, an alternative approach to combat *S. aureus* infections by limiting, during the colonization phase of the host by this microorganism, the availability of zinc, a metal that plays a key role in many metabolic processes (it is the second most abundant transition metal cofactor after iron). The authors demonstrated how zinc deprivation in vitro affects growth, biofilm formation, and the hemolytic activity of *S. aureus*. These findings may be a potential supportive approach to contain topical MRSA infections. 

Another manuscript by Hirakawa et al. investigated the role of major members of the outer membrane protein family OmpA, OmpW, and OmpX in the type III secretion system (T3SS)-mediated virulence of enterohemorrhagic *Escherichia coli* (EHEC) [12]. The authors showed that OmpA, but not OmpW and OmpX, contributes to the T3SS-associated pathogenicity of EHEC. These data significantly contribute to shedding light on the role of outer membrane proteins in bacterial pathogenesis.

This Special Issue also features a manuscript on the function of the MbovP0145, a protein present in the *Mycoplasma bovis* (*M. bovis*) secretome [13]. *M. bovis* is an important bovine pathogen responsible for huge economic losses in the dairy and beef industries worldwide. Previous studies have shown that the IL-8/neutrophil axis plays an important role in the pathogenesis of mycoplasma. The manuscript produced by Lu et al. shows that MbovP0145 induces the upregulation of IL-8 expression by interacting with beta-actin in the cytoplasm and contributing to activation of the MAPK signaling pathway. Thus, the increased expression of IL-8 is a crucial factor in the activation and migration of neutrophils from blood to the site of *M. bovis* infection. 

This Special Issue also features another manuscript that investigated a different aspect of *E. coli* pathogenesis, namely, the factors involved in the transition from intestinal commensal *E. coli* to invasive *E. coli* that causes bloodstream infections [14]. The work of Krall et al. provides evidence that the invasiveness of clinical isolates of *E. coli* from intestinal epithelial cells is dependent on the presence of an IncFII plasmid carrying the gene encoding for the protein modulating hemolysin expression (Hha). In addition, these authors showed that the transfer of the resistance plasmid IncFII, identified through an innovative survey approach that compares and analyzes clonal *E. coli* isolates from rectal swabs and blood cultures of the same individuals, contributes to the transition of commensal to invasive *E. coli* strains in vivo. 

This Special Issue also presents a review manuscript on the different functional aspects of Zur proteins found in various bacterial species [15]. The manuscript produced by Kandari et al. presents a detailed analysis and discussion of the roles played by the zinc uptake regulator (Zur) in the virulence of some pathogens and host–pathogen interaction. The authors of this review pointed out that not only is Zur a Zn-dependent transcriptional regulator, but it is also a master protein critical for the survival of many pathogens and may be a potential antimicrobial target.

Finally, to conclude this series, this Special Issue includes two review manuscripts dealing with the global problem of bacterial resistance to antibiotics and the advantages and limitations of molecular methods applied to the diagnosis of bacterial infections. The main mechanisms of antibiotic resistance in ESKAPE pathogens and a detailed description of the most commonly used classes of antibacterial drugs against these pathogens are critically reported in [16]. 

In the last review, Gerace et al. addressed the important issue of recent advances in the use of molecular methods for the diagnosis of bacterial infections and provided an overview of the possible range of applications of these methods [17]. The authors also highlighted the importance of developing methods to quickly and accurately identify MDR pathogens, an important goal not only for early initiation of appropriate antibiotic therapy but also for outbreak resolution.

Overall, this Special Issue highlights the complexity of pathogenetic mechanisms actuated by different bacterial species in the context of microbe–host interactions that impact both pathology and disease outcome. Understanding the pathogenetic mechanisms of these clinically well-documented phenomena is a priority for the development of novel and effective therapeutic approaches for disease prevention. The knowledge gained from the articles discussed here could contribute to the development of pathogen-specific strategies and significantly accelerate vaccine development.

We would like to thank all the authors who participated in this Special Issue and the reviewers for their helpful comments.
